# Patterns of and Rationale for the Co-use of Methamphetamine and Opioids: Findings From Qualitative Interviews in New Mexico and Nevada

**DOI:** 10.3389/fpsyt.2022.824940

**Published:** 2022-03-28

**Authors:** Brittany D. Rhed, Robert W. Harding, Charles Marks, Katherine T. Wagner, Phillip Fiuty, Kimberly Page, Karla D. Wagner

**Affiliations:** ^1^Division of Social Behavioral Health and Health Administration and Policy, School of Public Health, University of Nevada, Reno, NV, United States; ^2^Department of Internal Medicine, University of New Mexico Health Sciences, Albuquerque, NM, United States; ^3^Santa Fe Mountain Center, Santa Fe, NM, United States

**Keywords:** methamphetamine, opioid, rural, injecting drug use, drug smoking, Western U.S

## Abstract

**Introduction:**

Methamphetamine use and methamphetamine-involved deaths have increased dramatically since 2015, and opioid-related deaths now frequently involve methamphetamine. Nevada and New Mexico are states with elevated rates of opioid and methamphetamine use. In this paper, we report results from a qualitative analysis that examined patterns of methamphetamine and opioid co-use over participants' lifespan, factors that influence those patterns, and implications for health outcomes among users.

**Methods:**

Project AMPED was a multisite, mixed-methods study of methamphetamine use in Northern New Mexico and Northern Nevada. Between December 2019 and May 2020, qualitative interview participants were asked to describe their patterns of and reasons for co-administration of opioids and methamphetamine.

**Results:**

We interviewed 21 people who reported using methamphetamine in the past 3 months. Four primary patterns of methamphetamine and opioid co-use were identified: [1] using both methamphetamine and heroin, either simultaneously or sequentially (*n* = 12), [2] using methamphetamine along with methadone (*n* = 4), [3] using prescription opioids and methamphetamine (*n* = 1), and [4] using only methamphetamine (*n* = 4). Among those who used methamphetamine and heroin simultaneously or sequentially, motivations drew from a desire to enhance the effect of one drug or another, to feel the “up and down” of the “perfect ratio” of a goofball, or to mitigate unwanted effects of one or the other. Among those who used methamphetamine and methadone, motivations focused on alleviating the sedative effects of methadone.

**Conclusion:**

To address the emergent trend of increasing methamphetamine-related deaths, researchers, health care professionals, and community health workers must acknowledge the decision-making processes behind co-use of opioids and methamphetamine, including the perceived benefits and harms of co-use. There is an urgent need to address underlying issues associated with drug use-related harms, and to design interventions and models of treatment that holistically address participants' concerns.

## Introduction

On the heels of the 21st-century opioid overdose death crisis in the United States, a “fourth wave” of drug overdose deaths has emerged (1). Methamphetamine use and methamphetamine-involved deaths have increased dramatically, and opioid-related deaths now frequently involve multiple drugs, including methamphetamine (2–4). Some authors have discussed this phenomenon as a “twin epidemic” of opioid and methamphetamine-involved morbidity and mortality (5).

While recent surveillance data have focused national attention on the co-administration of opioids and methamphetamine, this phenomenon is not new. One of the most well-known patterns of co-administration, the simultaneous administration of heroin and methamphetamine in a single injection, colloquially referred to as a goofball, has been documented in numerous cities in the US and Mexico, principally in the West, since at least 2000 (6–9). More recent data suggest that the prevalence of this behavior may be increasing. For example, a 2019 study of syringe exchange program (SSP) clients in Seattle, Washington mentioned that 55% of participants reported using a goofball in the last three months, and the prevalence of reporting goofball as one's main drug increased from 10% in 2017 to 20% in 2019 (10). A 2017 study using National HIV Behavioral Surveillance (NHBS) data from Denver, Colorado, found that 28% of the sample reported using goofballs, 43.9% reported using heroin and methamphetamine separately, and 24% reported doing both (11). Demographic correlates of methamphetamine and opioid co-administration include younger age (9–11), experiencing homelessness (10–12), and recent incarceration (10).

Another common pattern of combining opioids and methamphetamine is the use of methamphetamine while using methadone (13–15). Globally, methadone maintenance therapy (MMT) is a predominant form of medication for opioid use disorder and it has been shown to help reduce injecting drug use, syringe sharing, engaging in risky sexual behaviors, and overall, the chances of HIV acquisition (13, 15, 16). In Iran, opioids are among the most frequently used substances, but methamphetamine has gained popularity. In 2013, Shariatirad et al. (17) documented co-use of methamphetamine among men enrolled in a methadone maintenance program; men said they did this to counter the sedative effects of the methadone, improve sexual performance, and increase energy. Finally, an Iranian study with women enrolled in a methadone program also reported frequent co-use of methamphetamine and heroin kerack (a high-purity synthetic heroin available locally), with 82/119 women (68%) reporting co-use (18). In a Chinese study, 13% of methadone clinic patients tested positive for methamphetamine and 9% tested positive for morphine and methamphetamine; methamphetamine use was associated with being on a higher dose of methadone (19).

The co-administration of methamphetamine and opioids, whether simultaneously or sequentially, can have important implications for the health and well-being of people who use drugs (PWUDs). This includes increased risk for overdose (6, 9, 11), and syringe sharing (9–11), which in turn increases the risk for bloodborne pathogen transmission (i.e., HIV, HCV) and soft tissue infections (10). In a study conducted in Denver, Colorado, participants who reported sequential use of heroin and methamphetamine more frequently reported an overdose in the past year, compared to those who reported injecting goofballs (38.9% vs. 20.7%) (11). Injecting both heroin and methamphetamine (vs. injecting only heroin) was associated with a 2.8 fold increase in the risk of past year overdose. In Seattle, Washington, participants who injected goofballs reported several high-risk injection behaviors, including neck injection, more frequent injection, public injection, and sharing injection equipment (10).

Less is known about how methamphetamine and opioid co-administration has evolved in other areas, including more rural Western states. Nevada and New Mexico are two mostly rural Mountain West states with elevated rates of opioid-related overdose death and prevalent methamphetamine use (3). Northern New Mexico, in particular, has had a long history of elevated opioid overdose deaths, a trend that preceded the current opioid overdose crisis across the US, and which has been characterized as a multigenerational phenomenon (20). Recently, however, anecdotal reports from harm reduction providers suggested that people who historically used heroin were initiating methamphetamine use. Nevada has also consistently ranked in the top quartile of opioid overdose deaths in the US, and the per capita rate of methamphetamine use was highest in the nation in 2018 (21). The objective of this paper was to identify and characterize patterns of methamphetamine and opioid co-use over participants' lifespan and to examine motivations and rationale for co-use (4). Implications of findings on better addressing the escalating overdose crisis and reducing harm related to methamphetamine and opioid use are discussed.

## Materials and Methods

### Recruitment and Data Collection

Data for the current study were collected as part of a larger sequential mixed-methods study (22). Between December 2019 and February 2020, we recruited people who use methamphetamine in Reno, Nevada and Rio Arriba County, New Mexico. Reno is a small city in northwest Nevada (population 250,000), located approximately 20 miles from the California border and 440 miles from Las Vegas in the southern part of the state. Rio Arriba County (population 40,000) is located in the north-central part of New Mexico, bordering Colorado. The closest major city is Santa Fe (~25 miles; population 84,000). Recruitment used a combination of street and agency-based outreach. This includes in-house recruitment at harm reduction/syringe services programs and street-based outreach in which outreach workers visited areas where PWUD congregate (e.g., homeless encampments, bus stations) to distribute flyers and inform people about the study. Three of the seven authors participated in outreach activities. We also conducted chain-referral recruitment through existing participants. Inclusion criteria were age 18 years and older and self-reported methamphetamine use in the past 3 months.

Trained qualitative interviewers used a loosely structured interview guide, which began with broad questions about the respondents' drug use, including reasons for using methamphetamine, current drug use patterns, and changes over time. Most relevant to the current analysis, we asked respondents to describe the context of their methamphetamine use, how their use began and how it has changed over time, how and when they use methamphetamine with or without other drugs, and what benefits and what drawbacks or negative experiences they are deriving from their methamphetamine use. Interviews were conducted in private or semi-private locations that were acceptable to the participants. Data were collected in English or Spanish, depending on participants' preferences. Written informed consent was obtained from all participants. A US$30 compensation was provided to all those who consented. All study activities were approved by the University of Nevada, Reno (UNR) Institutional Review Board (IRB). The University of New Mexico (UNM) IRB deferred oversight to the UNR IRB under a single IRB agreement.

### Analysis

Interviews were digitally recorded and transcribed verbatim for analysis. After conducting a quality assurance review and redaction of the transcripts, data were analyzed using an inductive thematic approach. An MPH-level analyst reviewed all the transcripts and began by making a series of memos documenting the drug use history and patterns reported by each respondent, including types of drugs used and routes of administration. Those memos were discussed with a study PI, a Ph.D.-level mixed methods researcher with 20 years of qualitative research experience, and together they began identifying dominant patterns of opioid and methamphetamine co-administration, based on the type of drug, timing, and route of administration of each drug. We categorized participants into mutually-exclusive groups that were based on the participants' most common pattern of co-administration. This was determined in one of two ways: [1] The participants stated a distinct preference, or [2] They talked predominantly about one pattern of drug use. For the purpose of this analysis, we have excluded 4 individuals who only used methamphetamine and focused only on those who report co-use of opioids and methamphetamine. Then, the analyst developed a set of codes that identified the rationale for using that way and perceived benefits and harms associated with each drug and route of administration. These codes were then systematically applied to each transcript. After an initial round of coding, the coded transcripts were reviewed and discussed with the PI, and codes were further defined and refined, while memos were expanded to include a description of each pattern. Finally, the analyst and the study PI discussed the output from the coding and identified illustrative quotes. Quotes are provided using respondent ID, ethnicity, race, sex, age, and location of the interview.

## Results

We examined patterns of co-use of opioids and methamphetamine among a sample of 21 participants from Northern Nevada (*n* = 11) and Northern New Mexico (*n* = 10). Respondents were 48% female and 40% Latinx. The median age was 35 years (IQR: 30–43). Just under half (48%) reported being homeless and 38% were employed full or part-time.

Seventeen of the 21 respondents (81%) reported co-administration of opioids and methamphetamine (four reported only using methamphetamine and were excluded from this analysis, as described above). We categorized participants into three primary groups of methamphetamine and opioid co-use patterns and identified patterns within each group. The groups include using: [1] both methamphetamine and heroin, either simultaneously or sequentially (*n* = 12); [2] methamphetamine and methadone (*n* = 4); [3] prescription opioids and methamphetamine (*n* = 1). However, it is important to note that most people had long-term histories of substance use and moved back and forth between different patterns of co-use within a given timeframe, so many people provided data about different patterns throughout their interviews. For example, Participant 4, a Hispanic/Latino Black/African American man in his 40's from New Mexico, began his drug use career snorting heroin. Subsequently, he switched to smoking heroin and began injecting methamphetamine, then switched to smoking methamphetamine. Now he is on methadone and smokes a “steady amount” of methamphetamine. Accounts of the transitions between patterns were particularly informative when identifying the motivations or rationales for preferring one pattern over another, which are discussed in the Materials and Methods section.

### Section 1: Patterns of Opioid and Methamphetamine Co-use

#### Group 1: Heroin and Methamphetamine

People whose preference was using methamphetamine and heroin represented the majority (*n* = 12; 71%) of our sample. Within that group, three sub-patterns were identified: simultaneous injection (i.e., “goofball”) or injection and smoking both drugs in quick succession, injecting heroin and smoking methamphetamine at separate times, and injection use of both drugs at separate times.

##### Simultaneous Injection or Injection and Smoking Both Drugs in Quick Succession (“Goofballs”)

“Goofballs” refers to the simultaneous use of methamphetamine and heroin. For most people, this meant combining methamphetamine and heroin in the same syringe which, when optimized, creates the “best of both worlds.” In the quote below, a respondent describes the “perfect ratio” that can be achieved. However, it is difficult for him to reliably achieve that perfect ratio, so he often uses them separately instead:

“Me and my girlfriend, we had shots together. She likes it together. *You do it right, you get a perfect ratio, I mean you can feel both of them. You get the high and then the low and then the high and then low*. But most times, you get one that just overpowers the other and then [it's] pointless for me. So I've tried to do it separate[ly].” –Participant 7, Non-Hispanic, White Male, 20's, Nevada. [emphasis added]

Some people liked the feeling of combining the drugs but preferred to smoke the methamphetamine immediately after a heroin injection, rather than injecting both. For example,

“I always said my favorite high would be shooting heroin and then smoking speed because you had energy, but you felt the effects of the heroin which I have always really liked.” –Participant 14, Non-Hispanic, White Female, 70‘s, Nevada.

##### Injecting Heroin and Smoking Methamphetamine Separately

Others specifically sought to *avoid* the effects of combining the drugs simultaneously, describing the undesirable effects of using goofballs. For example,

“I kind of feel like [a goofball] kills the euphoria from the heroin, so I would rather shoot the heroin and smoke the meth, like, something like that. [A goofball] just seems dangerous too. It's just consuming anything is bad enough, but shooting something, the two polar opposites. One's going up and down, your heart doesn't know what to do with it. I don't know which way to go. In my experience, they send me into kind of a psychotic break where I'm kind of screaming and lose all control.” –Participant 8, Non-Hispanic, multi-racial Male, 20's, Nevada.

For participants like this man, injecting heroin and smoking methamphetamine at separate times was preferred. He goes on to describe how he moderates his methamphetamine use over time:

“I want to say I control my limits with meth. It's not a daily thing anymore. After one or two days, I have to take a break because I just don't like going on days without sleep. It's just the mental side effects for me personally or just outrageous if [I'm] not careful with it.” –Participant 8, Non-Hispanic, multi-racial Male, 20's, Nevada.

##### Injection of Both Drugs at Separate Times

Finally, some respondents preferred to inject both heroin and methamphetamine at separate times (i.e., not as a combined shot or ‘goofball'). For example, Participant 3 described smoking methamphetamine as a “waste,” and explains why he prefers to inject methamphetamine separately:

“I do heroin and then do the meth and for some reason, it makes the heroin last longer, so you don't have to you know, you know what I mean? I don't know how or what. But that's for me anyways. I'd rather inject it… because when you smoke it, you waste some.” –Participant 3, Hispanic/Latino Male, 30's, New Mexico.

Participant 10 usually preferred goofballs, but sometimes injects heroin and methamphetamine separately, depending on his mood (which was also described by others):

I: Do you always mix them together or do you sometimes use [methamphetamine] separately?
P: Sometimes, separate. Just depends on my mood.
–Participant 10, Non-Hispanic, White Male, 30's, Nevada.

#### Group 2: Methamphetamine and Methadone

Three participants described occasional methamphetamine use while taking methadone as part of an OUD treatment program. These respondents had engaged in several cycles of OUD treatment (including both methadone and buprenorphine) and periods of returning to heroin use. While they were on methadone treatment, their methamphetamine use increased compared to when they were using heroin:

“*Now, it seems that I've been on the methadone, I smoke so much meth now* – *more than I ever have*. I kind of think because the methadone gets you so tired, so down.” … “What's amazing, to be honest with you, after I went on that trip, now that I smoke [meth], it's a whole different thing. I don't get high like that no more. Now, I'm normal. It just wakes me up a little. That's it. I don't trip. Thank God.” –Participant 15, Hispanic/Latina Female, 40's, New Mexico. [emphasis added]

The “trip” that this participant refers to was an experience of injecting methamphetamine that led to undesirable hallucinations. After that experience, she switched to smoking methamphetamine, which for her does not result in the undesired psychiatric effects.

Two respondents also described ongoing and occasional heroin use, in addition to their methadone and methamphetamine use. Participant 16, who is taking methadone as part of a treatment program but also continues to use heroin, methamphetamine, and cocaine, described the circumstances that lead her to choose one stimulant over another:

“With the coke, I guess, the coke is like if I just wanted a real quick wake up, just to wake up real quick. Then the meth, if I have a few things I have to do for like the next few days, it'll keep me up for the next few days and keep me going. So [meth] kind of gives the energy too a little bit more than the coke.” –Participant 16, Hispanic/Latina, Black/African American Female, 20's, New Mexico.

#### Group 3: Methamphetamine and Prescription Opioids

In one case, a respondent started her drug use with heroin and transitioned to buying prescription opioids on the street once she settled down and started a family with her husband. The switch to prescription opioids (in pill form) was precipitated by a desire to reduce the harms associated with heroin use. Subsequently, her husband introduced her to methamphetamine. They started by smoking methamphetamine but switched to injecting because it is easier to hide from other family members.

### Section 2: Motivations or Rationales: Pain, Pleasure, Function, Social Context, and Drug Availability

We identified three primary groups of opioid and methamphetamine co-users. Within each group, sub-patterns were described based on timing and route of administration of the drugs. Several factors influenced how participants made decisions about their preferred route of administration (i.e., injection vs. smoking) and sequence of co-administration, which we categorize into five themes: avoiding pain or discomfort, seeking pleasure, responding to social context, responding to drug availability, and achieving functional effects.

#### Avoiding Pain or Discomfort

Strategies to avoid pain or discomfort largely focused on the route and sequence of administration of opioids and methamphetamine, but also included using one drug or another to address specific pains or discomforts. For example, some people combined heroin and methamphetamine into a single goofball injection because they wanted to avoid the vein pain associated with multiple injections. For example, Participant 6 described a burning sensation associated with methamphetamine injection and a desire to “not poke twice”:

“My veins are so sore because I do maybe 5, 10, 15 shots a day. That's the worst part about it. It's the effects on my veins and the bruising. I really use the same site like these over and over if I can, but that only lasts for maybe a day. Then they're off and so I have to go find new ones again. Then it hurts bad. *And meth hurts. It burns bad. It burns. It hurts bad*. My veins are just so raw because of constantly doing it [injections]. Mainly, *I don't want to hit twice. I don't want to poke twice*. They just hurt so bad already. *I just want it [the heroin and meth] all at once*.” –Participant 6, Non-Hispanic, White Female, 30's, Nevada. [emphasis added]

Others who described vein pain and damage that they attributed to injecting methamphetamine switched to smoking methamphetamine to help avoid some of that pain. For example,

“Well, I shoot up heroin now and I used to shoot up meth until – *I mean [meth] messes up your veins so bad. It's ridiculous how fast it messes your veins up. I just smoke it now*. There's times where people offer me a shot. And if there's nothing else, I'll do it but I guess the chemicals or whatever that's in it. I mean it just – you go from having that vein there to not being able to hit it, it's just not having veins. And I always told myself I wouldn't be that person that was shooting up and taking hours and hours to hit but sometimes, I can't find a vein. *And the methamphetamine messed up my veins a lot. Not that the heroin didn't either but it's just faster – the meth messes with me*.” –Participant 5, Hispanic/Latina Female, 30's, New Mexico. [emphasis added]

Still, others described lung pain or potential for damage associated with *smoking* methamphetamine, and therefore preferred injecting it. For example, this person experienced unpleasant effects from smoking methamphetamine, which made her hesitant to try it again,

“When I first took a hit of meth, they didn't tell me not to hold it in like you hold in crack. So when I blew it out, my head started pounding. My friend was like, “Oh yeah. By the way, don't hold it in. You'll crystallize your lungs.” I was like, “Well, thanks.” So I had a migraine for two days after that. I didn't like it. So it took me a long time to try it [methamphetamine] again.” –Participant 5, Hispanic/Latina Female, 30's, New Mexico.

When she resumed methamphetamine use, she injected it to avoid the unpleasant effects of smoking that she experienced the first time.

As described earlier by Participant 15, many people mentioned that they experience undesirable psychiatric symptoms from injecting methamphetamine. As a result, they switched to smoking methamphetamine to avoid the adverse effects but still receive the pleasurable effects (e.g., increased energy). For example,

“I smoke it [meth]. I've only injected it a couple of times. It was too intense. When you inject it, it's more extreme. It hits you harder and it's more intense. Much faster and much harder. It hits you hard. Being almost erratic. It's just too much – breaking out in a cold sweat. *It's much better – smoking it – for me*.” –Participant 12, Non-Hispanic White Male, 50's, Nevada. [emphasis added]

Heroin-using respondents also described using methamphetamine to alleviate the pain and discomfort of opioid withdrawal:

“If you're doing meth, you're sort of up and running around. If you get sick on heroin [i.e., experience withdrawal symptoms] and you do meth, you really don't feel sick on heroin anymore for quite a bit. So you can get a lot of shit done like walk around and do whatever you're going to do.” –Participant 11, Hispanic White Male, 30s, Nevada.

#### Seeking Pleasure

A second theme concerned modifying the route or sequence of administration to enhance the pleasurable effects of one drug or the other. For those who prefer goofballs, the experience of injecting the drugs together was intensely pleasurable:

“I was doing it with meth and heroin. It's like, I don't know, it's a disgusting sort of pleasure. I don't know but it's hard to describe. But yes, I mix the two. Once you get to the point where you're like, and especially since I struggled, the pullback and you see the blood go back in the barrel and you know you're in a vein and when you press down, you're going to get a rush, nothing in this world will ever compare to that.” –Participant 11, Hispanic White Male, 30's, Nevada.

Participant 10, who also prefers goofballs, said:

I: So, the stuff that you're getting right now, how does it make you feel? In your body, what does it feel?
P: Oh, man. It's good. I do really large hits… But anyways, you fucking slamming here and then you flag it. The blood draws up back up in it. That's the fucking first time that I'm going to get fucking high as fuck. You feel it coming up your throat, you'll cough maybe, [inaudible] really good. It's fucking really good. It's really good. –Participant 10, Non-Hispanic, White Male, 30's, Nevada.

Several people reported that injecting methamphetamine results in the most intense effects. Although some people considered that intensity to be unpleasant (e.g., Participant 12 and 15, above), others actively sought out the intense pleasure. For example,

“It was bad, like, intense, like, knowing that that was what's supposed to happen. I would love to go back there but I was by myself in an apartment. I thought, “Oh, my god. I'm fucking going to die.” Yeah, it was really scary but knowing that that's what's supposed to happen. It's super, like, pleasure that you can never experience. I'm, like, coming to terms with the fact that there will never be a more purely pleasurable experience than that in my life. Orgasm, nothing, will ever compare to that. It's like a warmth and a sort of I don't know, weird headspace where sort of everything – it's strange to say. It's like your environment becomes erotic. Everything is sort of like a very – and later, I've realized, you actually like orgasm in your pants. You have a physical orgasm through injecting which... that sort of happened. I really put two and two together whereas now, I realize it's sort of like it's a sexual drug.” –Participant 11, Hispanic, White Male, 30's, Nevada.

Some people found the effects of methamphetamine more pleasurable when they smoked it. Other people simply enjoyed the act of smoking and described smoking methamphetamine to satisfy the urge they have to smoke. For example,

“Just smoking it [meth]. I was craving to smoke something, and cigarettes were – I was craving the smoke.” –Participant 2, Hispanic/Latina Black Female, 20's, New Mexico.

#### Responding to Social Context

Social context (e.g., family, especially partners) was also influential in determining the route and timing of co-administration. Although most people reported a distinct preference when it came to their pattern of co-administration, some people were flexible and would accommodate the preferences of the people they were with, even if it wasn't their preferred method. This was especially evident when people described using drugs with a partner or significant other. As previously mentioned, Participant 17, who used prescription opioids and methamphetamine, was heavily influenced by her husband's preferred drug (methamphetamine) and route of administration (injection), but continued using prescription opioid pills to address underlying issues. She was concerned about hiding her methamphetamine use from family members, so it was easy for her to switch from smoking to injection use (which she believed was easier for her to hide), especially since her husband was there to show her how to inject.

Participant 7, who earlier described the pleasure when a goofball achieves the “perfect ratio,” said he would rather do two separate injections of methamphetamine and heroin because, more often than not, that “perfect ratio” is not achieved and, as a result, one drug overpowers the other, rendering the injection “pointless” to him because he cannot feel the effects of the overpowered drug (e.g., too much methamphetamine and he cannot feel the heroin). When this happens, he finds himself spending more money on drugs or being miserable for the rest of the day if he is unable to afford more. Nevertheless, he uses goofballs with his girlfriend because that is her preferred method.

Finally, in the exchange between Interviewer (I) and Participant (P) below, the participant describes how his decisions about how to use were influenced by other members of his drug-dealing network:

I: Yeah. When you first used it, how did you use it? Did you smoke? Did you inject it?
P: I smoked it and I inject it, snort it. I just went all the first time –
I: Okay. Yeah. It's like all the different ones.
P: – because I've been a part of the cartel for a while. I mean when they offer you something, you have to do it. It's not like, “Oh, I don't want it. No, thanks.”
I: Yeah, just a little bit of like you've got to prove yourself.
P: Yeah. It's like a disrespect. If you don't do it, they'll think you're a drug or you're a narc or you're something.
–Participant 1, Hispanic Male, 40's, New Mexico.

#### Responding to Drug Availability

Another theme that influenced decisions about the route and timing of co-administration was drug or supply availability. Several respondents described specific preferences; however, they also described flexibility to adjust to changes in the availability and affordability of drugs and supplies (e.g., syringes). For those who preferred to use goofballs, they used them almost exclusively unless they could not afford it, leading to a hierarchy of drug use. This usually entailed using heroin first to avoid the negative effects associated with heroin withdrawal and then using methamphetamine once it became available. Participant 16 also described the declining quality of heroin, leading to a reduction in her use of that drug (while continuing to use methadone and methamphetamine). For those who preferred injection drug use, some reported resorting to smoking or snorting drugs if no (new, sharp) needles were available.

P: I mean I just smoke [meth], shoot it, it depends… It depends, like, what I have. If I have the syringes.
I: Yeah, okay. If you have syringes, if you have the equipment to inject it. Do you prefer one over the other? Smoking vs. injection?
P: I [would] rather inject it.
–Participant 3, Hispanic/Latino Male, 30's, New Mexico.

Finally, there were some respondents who had familiarity with cocaine, but for whom methamphetamine appeared to be a newer stimulant. For those respondents, they described learning the differences between methamphetamine and cocaine and adjusting their use accordingly (e.g., Participant 5 above describing having to learn not to hold in methamphetamine smoke). Below, Participant 4 describes his first injection of methamphetamine, which he thought would be like cocaine but instead sent him into a “spiral”:

“Methamphetamine, my first time using it was shooting it up, and then *it took me to a spiral*. I had no idea what it would – I thought it would be used like a – *I was told it was going to be the same thing as cocaine*.” –Participant 4, Hispanic/Latino, Black/African American Male, 40's, New Mexico. [emphasis added]

This respondent subsequently started smoking methamphetamine, which gave a less intense high, and enjoyed using heroin to help him calm down from the methamphetamine.

#### Achieving Functional Effects

Finally, participants described using methamphetamine in conjunction with opioids to achieve functional effects. These included “relaxing” with methamphetamine, using methamphetamine to counter the sedation of heroin or methadone, and coping with trauma.

Participant 4, whose primary pattern of co-use was smoking “a steady amount” of methamphetamine on top of his methadone, described the relaxing and calming effects he experiences from using heroin and methamphetamine. Importantly, he experienced undesired psychiatric effects from injecting the methamphetamine, and switched to smoking which gave him the desired effect:

“Now that I'm smoking [meth] with the bongs and the pipettes [rather than injecting it], I see myself – it does calm me down. It does allow me to – yes, *it does bring me into a calm mode*. It gets me into a place of relaxation, not too deprived of energy, not too deprived of less energy.” –Participant 4, Hispanic/Latino, Black/African American Male, 40's, New Mexico. [emphasis added]

Later, he went on to explain that the methamphetamine helps him stay busy, and he also uses heroin to relax and calm down from the busy-ness.

Others described using methamphetamine to “wake up” or “get energy” when they feel overly-sedated from heroin or methadone. For example:

“I used to inject heroin. It's gotten cut down because of the methadone use.”... “I believe that I use meth with heroin sometimes. Like I said, I get a little bit lazy on the heroin or the methadone and then *I want to come up and I want to start cleaning*, or I got to get energy to deal with stuff and I don't want to just be sleeping. *I'll take a puff to wake up or to get going*.” –Participant 15, Hispanic/Latina Female, 40's, New Mexico. [emphasis added]

Similarly, Participant 9 took heroin to address underlying pain, and used methamphetamine to give her more energy. However, she doesn't use methamphetamine without using heroin:

“I don't like to use meth without heroin. I don't know if it's because [it's] different nowadays. It is different, but I don't like the—it's like the heroin takes the edge off, because I like to be awake. I don't like to do a bunch of heroin. I don't like to be sleepy and stuff. I just like to—for one thing, I have pain but I don't have to do heroin. But I like doing the meth. It gives me energy”–Participant 9, Non-Hispanic White Female, 40s, Nevada

Participant 17, who started using methamphetamine when she switched from heroin to prescription pills, continued to use methamphetamine and occasional opioid pills because the methamphetamine helped her cope with long-term trauma, increased productivity and focus, and decreased tension with her partner.

“I would say it [methamphetamine] helped me deal with my trauma, I guess. I mean it's very escaping. I'm not clouded by any trauma that I've had in the past.”—Participant 17, Hispanic/Latina Female, 30's, New Mexico.

Finally, as described earlier, when participants had to make decisions about using heroin *or* methamphetamine (e.g., when they couldn't afford both), those who were dependent on heroin typically used heroin first, to ensure that they could avoid experiencing symptoms of heroin withdrawal.

## Discussion

We interviewed 21 people who use methamphetamine about their patterns of opioid and methamphetamine use. Notably, the majority (17/21, 81%) engaged in some form of co-administration. While we were able to identify dominant patterns, most respondents had a long history of drug use and had transitioned through many different combinations of timing, drug type, and route of administration. Within each of their preferred patterns of drug use, respondents described sub-patterns that were influenced by a complex set of motivations and rationales that sought to enhance some experiences (e.g., optimize pleasure) and reduce or mitigate others (e.g., avoid pain, counter over-sedation, etc.).

Our findings regarding the rationales underlying patterns of co-use are like those identified in other areas, including Melbourne, Victoria, Australia, and Oregon, USA. Palmer et al. (23) conducted a qualitative study in Melbourne, Victoria, Australia (population 5 million) and identified three main reasons for co-administration: using one drug to balance or manage the negative side effects of the other (in their case, using opioids to treat the effects of coming down from a methamphetamine binge), using one drug to enhance the effects of the other (in their case, using methamphetamine to prolong heroin intoxication or combining the two because it “feels better”), and using methamphetamine to get “high” while using a form of MOUD. Ellis et al. (5) found that 51% of their sample of 145 key informants endorsed the “high seeking” reason for co-administration, 39% endorsed the “balancing” rationale, and 15% reported using methamphetamine as an “opioid substitute.” Our findings reflect very similar rationales, with most of our respondents describing co-administration of heroin and methamphetamine as a way to enhance or optimize the desirable sensations of both. Radfar et al. (24) identified a high prevalence of methamphetamine use among methadone maintenance patients in Iran. Many reported that the effects of methadone and methamphetamine were *better* than the effects of using methamphetamine with other opioids, such as heroin or opium. Our findings extend this knowledge by also describing drug-by-route interactions, such as attempting to avoid pain specifically associated with injecting or smoking methamphetamine to reduce undesirable effects and increase pleasurable ones.

The social context of use is also an important determinant of both drug use patterns and related harms (25). For example, a large body of research describes the outsized influence played by female PWID's male sexual partners in structuring their initiation into and ongoing access to drugs (26, 27). We observed similar situations here, in which women were introduced to methamphetamine use by their male partners. However, this gender dynamic is not unidirectional (28); we also observed a male PWID adjusting his preferred pattern based on his female partner's preferences. Other social considerations included a woman's decision to inject methamphetamine rather than smoking it, as a way to hide her use more effectively from others in her household, and a man's decision that was influenced by members of his drug-selling network.

In terms of availability, sometimes drug availability made one's first choice unobtainable, in which case they would resort to using what they could obtain. In these cases, attending to opioid withdrawal symptoms became a priority, with the stimulant effect of methamphetamine being secondary (but sometimes also an attempt to deal with the opioid withdrawal, if no heroin was available). We also observed discussion of the changing (typically declining) quality of drugs over time, and the influence of quality on consumption patterns. It may be that changes in methamphetamine composition could also underlie some of the experiences described in this study. Specifically, methamphetamine containing *d*-methamphetamine salts without *l*-methamphetamine salts, which can be removed during some manufacturing processes (29), has been associated with stronger and shorter duration effects, a “sleepy” effect (sometimes described as “shutting down”) after using methamphetamine (22), and more psychiatric symptoms such as delusions and paranoia (30), some of which were described by our respondents as undesirable effects they sought to mitigate.

Finally, it is important to note the functional nature of the drug use patterns described by our respondents. Several studies have noted that function is a salient dimension of methamphetamine use, with people reporting increased ability to meet everyday tasks, better focus, and increased productivity (31) and to increase income generating ability (32). Others have explored motives for stimulant use using domains such as: enhancement, coping, social, and conformity (33, 34). While these studies did not assess motives for co-administration of methamphetamine with opioids, there are similarities in this study's findings. People described several needs (i.e. to avoid withdrawal symptoms, to wake up, to treat trauma, to counter the sedation from methadone) that were met by their drug use, and opioids and methamphetamine served different functions. Our findings do correlate with a recent examination of co-administration of drugs among methadone maintenance treatment patients, which showed that enhancement (seeking pleasure or to get high) was the primary motive (35). Radfar et al. (24) also identified several reasons for using methamphetamine while on methadone. These reasons include coping with conflict and stress, tolerating undesirable effects of methadone (e.g., lethargy, sexual dysfunction), and self-management of opioid (and other drug) cravings while on methadone maintenance. The overwhelming majority of participants in that study indicated that methamphetamine use is normative among patients and that methamphetamine use is encouraged within social circles as a way to combat the side effects of methadone during the early stages of methadone treatment.

### Implications: Harm Reduction and Trauma-Informed Approaches

There are several health-related implications to consider from our research. First, combining stimulants with opioids is a well-established risk factor for opioid overdose death. Not only did our participants describe combining heroin or prescription opioids with methamphetamine, but several also described using heroin, methamphetamine, *and* methadone. Concerningly, a recent study (36) found that most respondents in their study in Dayton, Ohio believed that methamphetamine could be used as a preventive measure to reduce the risk of opioid overdose in a fentanyl-saturated market, or administered as a last resort to reverse the effects of an opioid overdose (especially when naloxone was not available). Combined, these findings reinforce the ongoing imperative to ensure that PWUD are properly trained in overdose recognition and response, and have naloxone available at every drug use event. Overdose prevention efforts should cast their net broadly and include people who use methamphetamine. Another risk factor for overdose is changing the route of administration (i.e., moving from smoking to injection). We found that people transitioned between smoking and injection for several reasons, including pain, vein damage, social context, and to increase or decrease the effects of the drug. Incorporating messages about how the route and sequence of administration can impact overdose risk and providing lower-risk options for administration could be an important addition to existing overdose prevention efforts.

Injection drug use is also a risk factor for transmission of HIV, hepatitis C virus, and other bloodborne and soft tissue infections if a sterile syringe is not used. Importantly, respondents in our sample nearly always described drug use in a social context. While using drugs in the presence of others is protective against overdose death (because someone will be there to observe and respond to the overdose), it can elevate the risk for pathogen transmission if people do not have access to enough sterile injection supplies. While some respondents in our sample reported smoking (rather than injecting) when sterile supplies were unavailable, it is important to note that our respondents were recruited from communities with fairly robust syringe services programs. This may not be the case for communities throughout the Mountain West, and overall, the US has not yet achieved sufficient syringe supply to allow PWIDs to use a new, sterile syringe for each and every injection.

Our findings suggest the potential importance of employing theoretical frameworks that can capture the functional (often ameliorative or protective) motivations for drug use, as well as associated risks and harms. The Trauma-Informed Theory of Individual Health Behavior (TTB) provides such a lens (37). Instead of conceptualizing drug use as inherently and exclusively harmful, it suggests that drug use can confer both protection and harm, dependent on context. TTB highlights that individuals make the best effort to address the most immediate harms that they are facing, and that attempts to change health behaviors must address an individual's focus on these immediate concerns before attempting to change future behavior (26). While we have identified some health harms (e.g., the risk for overdose and bloodborne pathogen transmission), our findings also support the idea that individuals use different drugs to alleviate or otherwise address specific sources of harm they are exposed to (e.g., untreated pain, income instability). Intervention and treatment efforts should consider that people who use drugs may have underlying needs that are currently being addressed via their drug use—therefore identifying and helping PWUDs address those underlying needs must precede attempts to change other health behaviors. For example, the use of methamphetamine to counter the sedative effects of methadone suggests that PWUDs on methadone maintenance might benefit from conversations with healthcare providers about the appropriateness of their methadone dose, and TTB would suggest that this needs to happen before efforts to reduce methamphetamine use. While only described by one of our respondents, the use of methamphetamine to self-medicate underlying trauma suggests that individuals with histories of trauma would benefit from comprehensive behavioral health care. Indeed, other research has shown that motivations for the co-administration of methamphetamine and opioids are complex and multifaceted, influenced by an abundance of personal and social factors that have an influence on drug use behaviors (5, 38). A recent study by Silverstein et al. (39) conducted in Ohio supports the idea that methamphetamine use among people who use opioids depends not only on individual and social factors, but also historical and pharmacological contexts that have implications for health outcomes and drug use trajectories. A TTB-informed approach would make explicit and address those underlying factors before attempting to change other health behaviors.

### Limitations

Our results should be considered in light of the study's limitations. Qualitative methods are designed to elicit a diversity of narrative descriptions, rather than generalizable conclusions, and therefore the findings from this study may not be transferable to other regional, cultural, or social settings. Specifically, both New Mexico and Nevada have experienced a high prevalence of methamphetamine use for decades, which may suggest that co-use patterns in these states differ from the Eastern US and other regions where methamphetamine use is more novel. However, the similarity of our findings with other quantitative and qualitative studies suggests that our conclusions are robust to such variation. We arranged people into their respective groups based on either their stated preferences or their most predominantly discussed patterns of use. However, this does not encapsulate the whole picture because people engaged in several patterns of polysubstance use over time. Responses may be subject to social desirability bias, in which respondents alter their responses based on what they believe is acceptable to the interviewers. Interviewers for this study were trained to mitigate such bias, and were embedded within the harm reduction infrastructure in each community to enhance relationships and facilitate open and honest dialogue with respondents.

## Conclusion

We identified dominant patterns of opioid and methamphetamine co-use and described motivations that influenced the type of drugs used, timing, and route of administration. Findings suggest that respondents engaged in opioid and methamphetamine co-use to address a number of underlying issues and unmet needs, and their patterns of use changed in response to social conditions and drug availability. Patient-centered models of care and support should seek information from participants about their unique drug use patterns, including their motivations for use and the needs currently being met by their use, and interventions and programs should holistically address participants' concerns. Polydrug use, in particular, is understudied and the motivations for co-administration of methamphetamine and opioids needs further inquiry. One potential theory for the development of more patient-centered understandings of methamphetamine and opioid co-administration is the Trauma-Informed Theory of Individual Health Behavior (TTB) (37), which explicitly addresses the underlying individual, social, and structural drivers of substance use behavior.

## Data Availability Statement

Because of the sensitive nature of the information contained in the transcripts (e.g., details about illegal behavior) and potential for severe ethical, legal, and social consequences resulting from broken confidentiality, full transcripts will not be made publicly available. Redacted excerpts of the qualitative transcripts used in the current analysis will be made available to qualified researchers subject to review and approval by the appropriate Institutional Review Board(s). Requests can be made to the University of Nevada, Reno Research Integrity Office by calling +1-775-327-2368.

## Ethics Statement

The studies involving human participants were reviewed and approved by University of Nevada, Reno (UNR) Institutional Review Board (IRB). The patients/participants provided their written informed consent to participate in this study.

## Author Contributions

We use the Contributor Roles Taxonomy (CRediT) refined by the Consortia Advancing Standards in Research Administration (CASRAI) for describing authorship contributions. BR and KDW: formal analysis. CM and KDW: writing—original draft. RH and KTW: project administration and investigation. KP and KDW: funding acquisition and supervision. RH, KTW, KP, and KDW: conceptualization and writing—review and editing. All authors contributed to the article and approved the submitted version.

## Funding

This research was provided by the Mountain West Clinical & Translational Research Infrastructure Network, in the form of a Multi-Site Pilot Project, through grant NIH/NIGMS U54GM104944; PI: Francisco Sy, MD, DrPH.

## Conflict of Interest

The authors declare that the research was conducted in the absence of any commercial or financial relationships that could be construed as a potential conflict of interest.

## Publisher's Note

All claims expressed in this article are solely those of the authors and do not necessarily represent those of their affiliated organizations, or those of the publisher, the editors and the reviewers. Any product that may be evaluated in this article, or claim that may be made by its manufacturer, is not guaranteed or endorsed by the publisher.
